# Massive hemorrhage after percutaneous kidney biopsy caused by renal artery malformation: a case report and literature review

**DOI:** 10.1186/s12893-020-00918-1

**Published:** 2020-10-29

**Authors:** Dong Liang, Hui Zhang, Min Yang, Hong Ji, Gang Chen, Ning Yu, Xiaomin Zhang

**Affiliations:** 1 Department of Nephrology, Binzhou Medical University Hospital, No. 661 Huanghe Second Road, Binzhou, 256603 Shangdong P.R. China; 2 Department of Pathology, Binzhou Medical University Hospital, No. 661 Huanghe Second Road, Binzhou, 256603 Shandong P.R. China; 3 Department of Vascular Intervention, Binzhou Medical University Hospital, No. 661 Huanghe Second Road, Binzhou, 256603 Shandong P.R. China

**Keywords:** Kidney, Biopsy, Accessory renal artery, Hemorrhage, Embolization

## Abstract

**Background:**

Accessory renal artery (ARA) is the most common site for anatomical variation of renal supply artery. Rare studies reported interventional embolization for the management of massive hemorrhage caused by ARA injury after percutaneous kidney biopsy (PKB).

**Case presentation:**

We describe a 35-year-old man who developed massive hemorrhage after PKB leading to shock. Digital subtraction angiography (DSA) showed hemorrhage in the ARA at the inferior pole of the right kidney and hemostasis was noticed after renal artery embolization.

**Conclusions:**

We proposed that much attention should be paid to the presence of ARA before PKB. In addition, digital subtraction angiography combined with superselective embolization is the best choice for the treatment of renal artery injury.

## Background

Percutaneous kidney biopsy (PKB) is the primary diagnostic tool for the determination of pathological type and severity of renal disease. In clinical settings, PKB has been well accepted by the majority of patients as it is mini-invasive. However, many patients present renal injury, hematuria, perirenal hematomas, arteriovenous fistulas and infection afterwards. Rare cases show life-threatening massive hemorrhage.

Accessory renal artery (ARA), a functional terminal vessel entering the kidney directly or via the renal hilum, shows the highest incidence of anatomic variation in the renal arteries. The presence of ARA can significantly increase vascular and urologic complications during renal surgery [1, 2]. To our best knowledge, rare studies reported massive hemorrhage caused by ARA injury during or after renal biopsy. In this study, we presented a case underwent PKB with massive renal hemorrhage due to ARA injury.

## Case presentation

A 35-year-old man referred to our department due to urinary abnormalities in January 2012. He showed no fever, joint and muscle pain, rash, diarrhea, lymphadenopathy, malar rash, oral ulcer, lumbar pain, photosensitivity or arthralgia, headache, chest tightness, suffocation, or palpitation. The heart rate was 82 beats per minute, and respiratory rate was 20 breaths per minute. The blood pressure was 154/95 mmHg. The laboratory findings were as follows: white blood cells (WBCs), 5.22 × 10^9^/L; hemoglobin (Hb) 125 g/L, red blood cells (RBCs), 4.08 × 10^12^/L; platelet 2.96 × 10^11^/L; albumin, 59.7 g/L; blood urea nitrogen, 5.27 mmol/L; C4, 18.7 mg/dL; C3, 118 mg/dL; and creatinine, 79.6 μmol/L. The hepatitis b surface antigen, anti-hepatitis B e antibody and anti-hepatitis B core antibody were positive. The serum HBV DNA level was 1.28 × 10^3^ IU/mL. There was urine blood (+ +) and protein (+ +). The urine total protein was 0.74 g/24 h. There were negative findings in the tests of anti-nuclear antibody (ANA), antineutrophil cytoplasmic antibodies (ANCA), anti-DNA antibodies, anti-myeloperoxidase (MPO) and proteinase 3 (PR3) antibodies, anti-glomerular basement membrane (anti-GBM) antibodies and anti-Streptolysin O. To identify the cause of urinary occult blood and the existence of hepatitis B associated glomerulonephritis, the patient was recommended to receive renal biopsy.

**Fig. 1:**
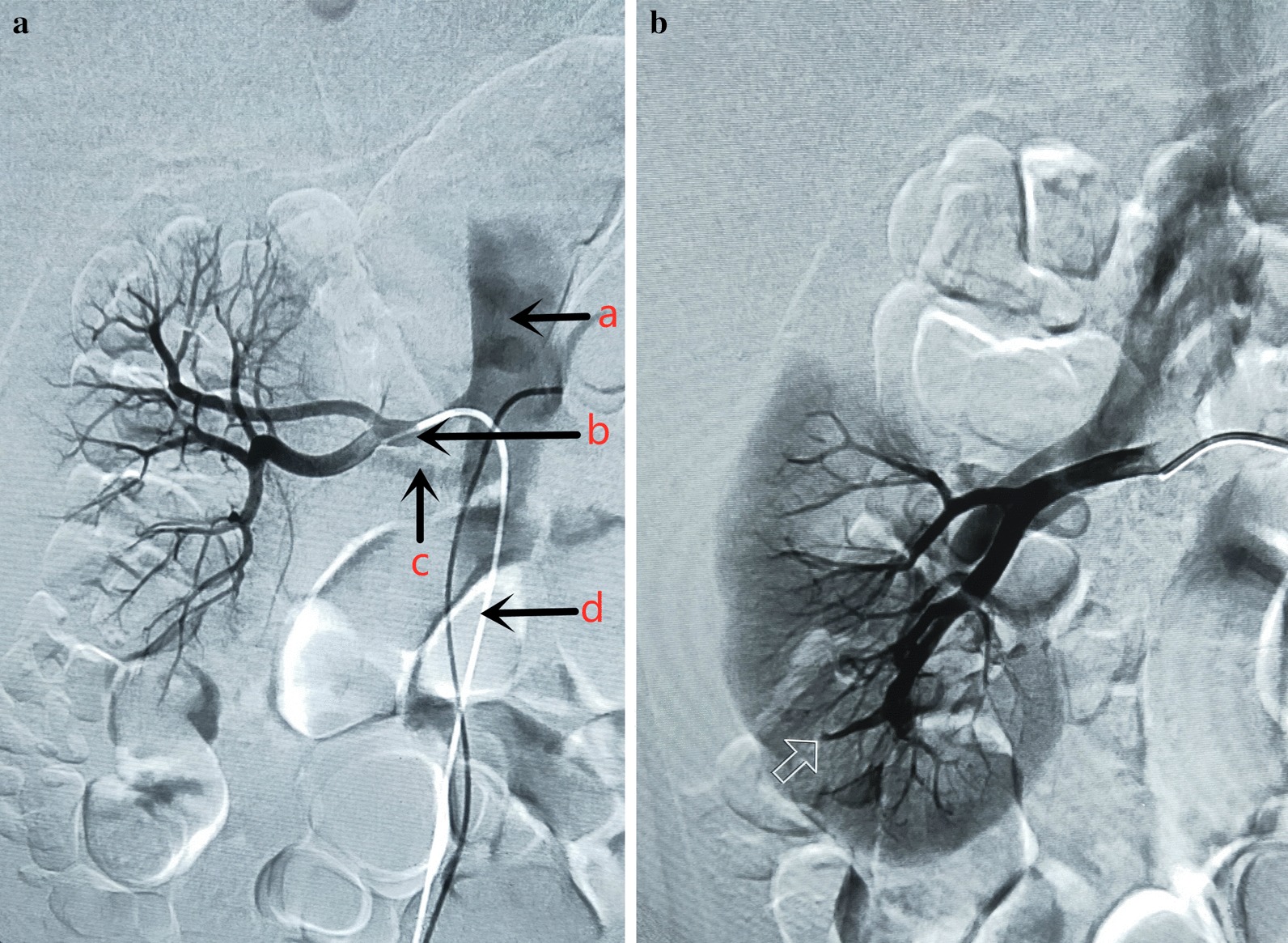
Image of right kidney by DSA. **a** Angiography from main renal artery. (a) Abdominal aorta; (b) Right renal artery; (c) accessory renal artery (ARA); (d) Interventional catheter. **b** Angiography from ARA. The arrow pointed to the site of arterial injury caused by the biopsy needle

**Fig. 2:**
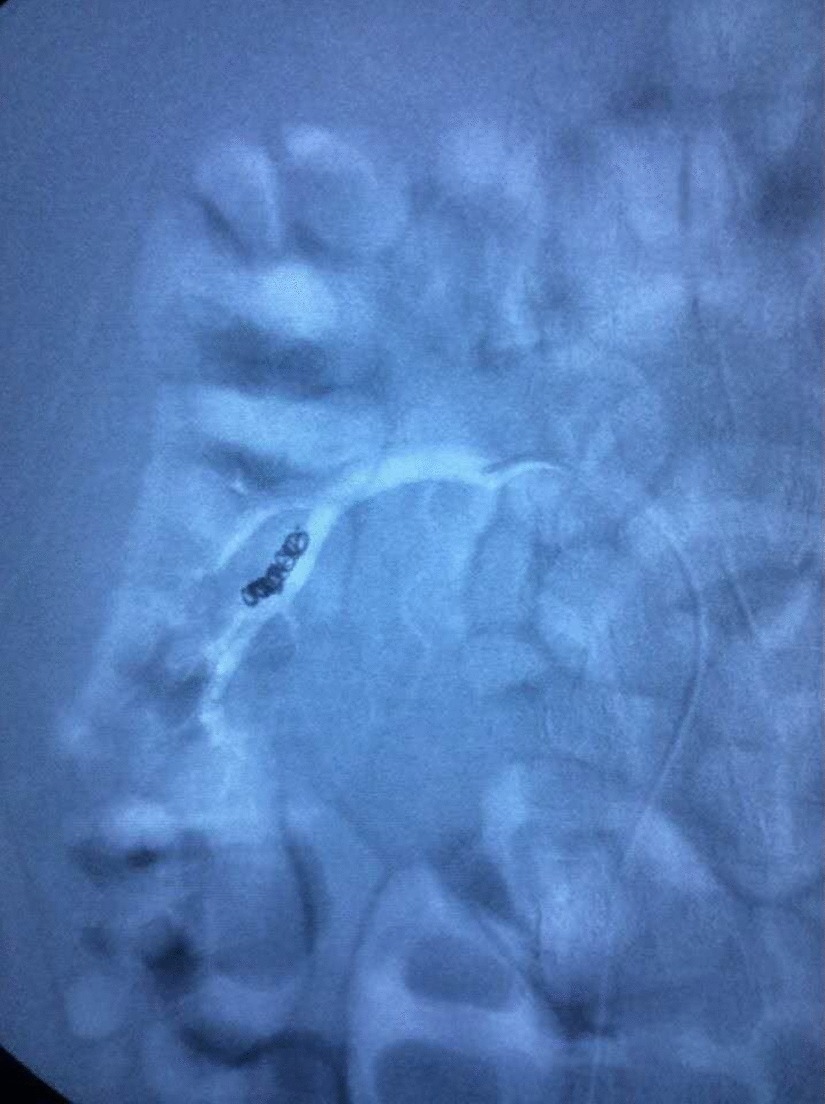
Hemostasis was obtained in the ARA at the inferior pole of the right kidney after vascular embolization with coil and gelatin sponge particle

Tru-cut puncture needle (16-gauge) was inserted in the lower pole of the right kidney for 3 times, and the puncture point was pressed for at least 5 min after renal biopsy. Afterwards, cefthiamidine (2.0 g) was given via intravenous drip to prevent infection. The patient presented no obvious discomfort during and after the biopsy. About 4 days later, he showed sudden shock after strenuous exercise with a systolic blood pressure of 80 mmHg accompanied by gross hematuria. Then the patient was carefully checked. The patient was required to rest in bed, and was immediately given hemostasis, continuous catheterization, and fluid replacement therapy. The catheterization led to a dark red color with blood clots (600 mL). Meanwhile, the ultrasound of the urinary system showed no obvious changes in the size and shape of the right kidney, and the collection system was separated by 1.1 cm. The bladder cavity had a low echo, suggesting a blood clot. Combined with the renal biopsy, renal hemorrhage was considered. After 2 days of conservative treatment, the patient showed dizziness, chest tightness with throbbing pain in the right waist, decrease of blood pressure to 59/36 mmHg. The Hb was 82 g/L. Considering the bleeding and rapid decrease of hemoglobin, the patients received infusion of concentrated red cell. Ultrasonography showed that a 2.9 cm × 2.2 cm × 2.0 cm low echo considering the blood clot in the right renal pelvis, and a low echo (14.2 cm × 8.0 cm × 9.1 cm) was considered blood clots in the bladder. After continuous catheterization, there was still light red fluid with waist pain. ECG monitoring showed a blood pressure of 110/65 mmHg, heart rate of 85 bpm and Hb of 54 g/L. In the presence of active bleeding, he was transmitted immediately to the Intensive Care Unit (ICU), where interventional embolization treatment was given. There was hemorrhage in the ARA at the inferior pole of the right kidney (Fig. 1). The hemorrhage was terminated after embolization (Fig. 2).

Renal biopsy indicated IgA nephrology. It was found that the punctured kidney tissue included cortical tissue, cortical medulla junction tissue and medulla tissue, of which about 10% of the medulla tissue. Based on the Oxford classification for the IgA nephropathy [3], the score was M1E0S1T1C0. Finally, the patient was discharged with no sequela. In the 1-year follow-up, there was no gross hematuria or signs of renal insufficiency.

## Discussion

Renal pathology is critical for the establishing of treatment plan and evaluation of prognosis among patients with renal disease. To date, PKB is considered to be an irreplaceable tool for the diagnosis, prognosis, and treatment of a variety of renal diseases. Despite the presence of a variety of new techniques, hemorrhage is inevitable after PKB [4–6]. Hemorrhage is considered the most common postoperative complication. The most significant complication of PKB is vascular damages with subsequent hemorrhage [7]. To date, there are many cases with injuries in lumbar artery and extrarenal artery, including mesenteric artery, intercostal artery and abdominal aorta. However, few reports focused on the hemorrhage caused by ARA injury after renal biopsy. A comprehensive literature research was conducted to the PubMed database and EMbase using the following key words: accessory renal artery; hemorrhage. Only three cases including our case were found [8, 9] (Table 1).

**Table 1:** Cases with hemorrhage after PKB caused by accessory renal artery injury

Author (year)	Basic conditions			Initial clinical diagnosis	Involved artery	Origin of the injured artery	Pathological diagnosis	Treatment	Prognosis
	Age, years	Gender	Basic diseases						
Harada et al. [8]	Male	24	Obesity	Obesity-related nephropathy	Aberrant renal artery at the lower pole of the right kidney	Aorta	Benign nephrosclerosis with secondary focal segmental glomerulosclerosis	Transarterial embolization	Hemostasis and complete remission of proteinuria
Zhang et al. [9]	Male	67	Hypertension	Nephrotic syndrome	Aberrant renal artery at the lower pole of the right kidney	Not available	AL-type renal amyloidosis	Renal arteriography and vascular embolization	Hemostasis
This case	Male	35	HBsAg, Anti-HBe, and Anti-HBc positive	Hepatitis B associated glomerulonephritis, suspected	Aberrant renal artery at the lower pole of the right kidney	Aorta	IgA Nephrology	Superselective renal artery embolization	Hemostasis

Arteries entering the kidney are divided into main and the ARA. Compared with the renal artery, the ARA started from the starting point showed smaller internal diameter than that of the renal artery. The path was longer, and its perfusion pressure was low. The blood flow resistance was high, and it was more prone to stenosis and bleeding. The incidence of ARA in Chinese is 14.5% [10], which varies in different countries, populations, genders, left and right sides. According to the previous studies [11, 12], ARA can originate from various arteries, including abdominal aorta, aortic and renal artery, external ilium artery, lumbar artery, as well as spleen artery. The most common variation of the renal artery was originated from the abdominal aorta, and the inferior renal artery was the most common type of variation. The lateral visceral artery of the embryo did not completely disappear during the development of the human body, which formed the ARA. This represented the traces of the blood supply of the kidney and embryo. The ARA was complex in origin, course, and position of entering the renal hilum, which increased the complexity of renal vascular anatomy and the difficulty of related surgical operation. In addition, the existence of the ARA was potentially associated with renovascular hypertension [13] and hydronephrosis [14].

For the patients with a small dimension of main renal artery by ultrasonography, there may be an ARA [15]. There was a certain diagnostic rate for ARA by ultrasonography and a high rate of misdiagnosis and missed diagnosis due to obesity or bowel gas. However, ultrasonography could be used as a preliminary screening test for ARA with features of convenient operation, repeatability and non-invasiveness. Unfortunately, the ARA can be easily misdiagnosed as renal artery stenosis solely based on the size of the diameter. As a non-invasive examination, CTA can clearly show the ARA with a diameter of more than 0.5 mm. In addition to clearly displaying the diameter of the renal artery, it can also show the number and shape of the renal artery, which can be utilized as a standard for evaluating the preoperative anatomical structure of the renal artery [16]. Digital subtraction angiography (DSA) is the gold standard for the diagnosis of renal vascular malformations. It is superior in displaying the branches of small arteries and evaluating the capacity of multiple supply vessels. In clinical practice, the non-invasive imaging methods are more likely to be accepted by patients, such as computed tomography angiography (CTA), and color Doppler ultrasound. Therefore, ultrasonography is suitable for primary screening and iodine allergy, renal failure, and pregnancy. Taken together, CTA can be used for routine examination, while DSA can be used for the final diagnosis.

Superselective renal artery embolization was regarded as a safe and effective treatment strategy for hemorrhage after PKB. For the patients with no signs of hemorrhage after renal arteriography, lumbar or iliolumbar arteriography is necessary [17]. Some patients may present complications after renal artery embolism, including post-embolism syndromes, infection and renal insufficiency [18], despite a lower incidence of renal artery embolism. The post-embolism syndrome is characterized by fever and lower-back pain. After symptomatic treatment, the complications after embolization were attenuated without special treatment. In our case, he showed no significant complications after embolization in the follow-up (Table 2).

**Table 2:** Patient blood pressure and related laboratory data

	On admission	Before operation	3 days after operation
Blood pressure (BP)	154/95 mmHg	110/65 mmHg	122/73 mmHg
Heart rate (HR)	82 bpm	87 bpm	80 bpm
Hemoglobin (Hb) (110 g/L–160 g/L)	125 g/L	54 g/L	78 g/L
Platelet (PLT) (1.0 × 10^11^/L–3.0 × 10^11^/L)	2.96 × 10^11^/L	2.6 × 10^11^/L	3.18 × 10^11^/L
Prothrombin time (PT) (11S–15S)	14.0S	13.6S	-
Activated partial thromboplastin time (APTT) (28S–34S)	38.0S	34.6S	-
International normalized ratio (INR) (0.8–1.5)	1.09	1.01	-
Blood urea nitrogen (BUN) (3.9 mmol/L–7.1 mmol/L)	5.27 mmol/L	4.13 mmol/L	5.34 mmol/L
Serum creatinine concentration (sCr) (0 μmol/L–132 μmol/L)	79.6 μmol/L	101.3 μmol/L	74.1 μmol/L

- Means not detected

In this case, we realized that iatrogenic renal hemorrhage should be considered when there were a serious hematuria and shock symptoms after renal biopsy, in which ARA injury was one of the causes. For the treatment of iatrogenic renal hemorrhage, routine medical conservative treatment was the first choice and then interventional therapy or surgical treatment must be considered. Medical treatment included absolute bed rest, ECG monitoring, recording the color and total amount of drainage, intravenous application of hemostatic drugs, continuous catheterization, as well as fluid replacement. At the same time, blood routine examination should be regularly reviewed, blood coagulation function should be monitored, and plasma or concentrated red blood cells should be infused if necessary. While ensuring the body temperature, correct the possible acidosis and maintain the stability of the internal environment. Patients with no responses to conservative treatment should be treated with interventional or surgical treatment in time.

## Conclusions

The presence of ARA may be one of the factors that increase the risk of bleeding in renal biopsy. For the patients underwent PKB, the color Doppler ultrasonography should be perfected before the operation. In addition, attention should be paid to the possibility of abnormal renal arterial hemorrhage. Suspicion for ARA injury with active hemorrhage is a definite indication for CTA or DSA. Moreover, superselective renal artery embolization may be the best treatment option.

## Data Availability

The data were available upon appropriate requests.

## Abbreviations

ARA: Accessory renal artery
PKB: Percutaneous kidney biopsy
DSA: Digital subtraction angiography
CTA: Computed tomography angiography
